# Nanohydroxyapatite-Protein Interface in Composite Sintered Scaffold Influences Bone Regeneration in Rabbit Ulnar Segmental Defect

**DOI:** 10.1007/s10856-022-06657-4

**Published:** 2022-04-09

**Authors:** Janani Radhakrishnan, Manjula Muthuraj, Gnana Santi Phani Deepika Gandham, Swaminathan Sethuraman, Anuradha Subramanian

**Affiliations:** Tissue Engineering & Additive Manufacturing (TEAM) Lab, Center for Nanotechnology & Advanced Biomaterials, ABCDE Innovative Centre, School of Chemical & Biotechnology, SASTRA Deemed University, Thanjavur, 613 401 India

## Abstract

The healing physiology of bone repair and remodeling that occurs after normal fracture is well orchestrated. However, it fails in complex clinical conditions and hence requires augmentation by grafts. In this study, composite nanohydroxyapatite (nHA), poly(hydroxybutyrate) (PHB) and poly(ɛ-caprolactone) (PCL) constituted microspheres sintered three-dimensional scaffold were evaluated in rabbit ulnar segmental defect. A composite scaffold using PHB-PCL-nHA microspheres was developed with protein interface by solvent/non-solvent sintering to provide multiple cues such as biocomposition, cancellous bone equivalent meso-micro multi-scale porosity, and compressive strength. In vitro DNA quantification and alkaline phosphatase (ALP) assays revealed that the protein interfaced composite scaffolds supported osteoblast proliferation and mineralization significantly higher than scaffolds without protein and TCPS (*p* < 0.05). Scanning electron micrographs of osteoblasts cultured scaffolds demonstrated cell-matrix interaction, cell spreading, colonization and filopodial extension across the porous voids. Cylindrical scaffolds (5 × 10 mm) were implanted following segmental defect (10 mm) in rabbit ulnar bone and compared with untreated control. Radiography (4, 8 and 12 weeks) and µ-computed tomography (12 weeks) analysis showed directional bone tissue formation by bridging defective site in both scaffolds with and without protein interface. Whereas, undesired sclerotic-like tissue formation was observed in control groups from 8 weeks. Histology by hot Stevenel’s blue and van Gieson’s picrofuchsin staining has confirmed enhanced bone maturation in scaffold groups while presence of osteoids was observed in control after 12 weeks. Thus, the developed composite matrices exhibits osteoinductive, osteoconductive properties and demonstrates its bone regenerative potential owing to its compositional, micro & macro structural and mechanical properties.

**Figure:**
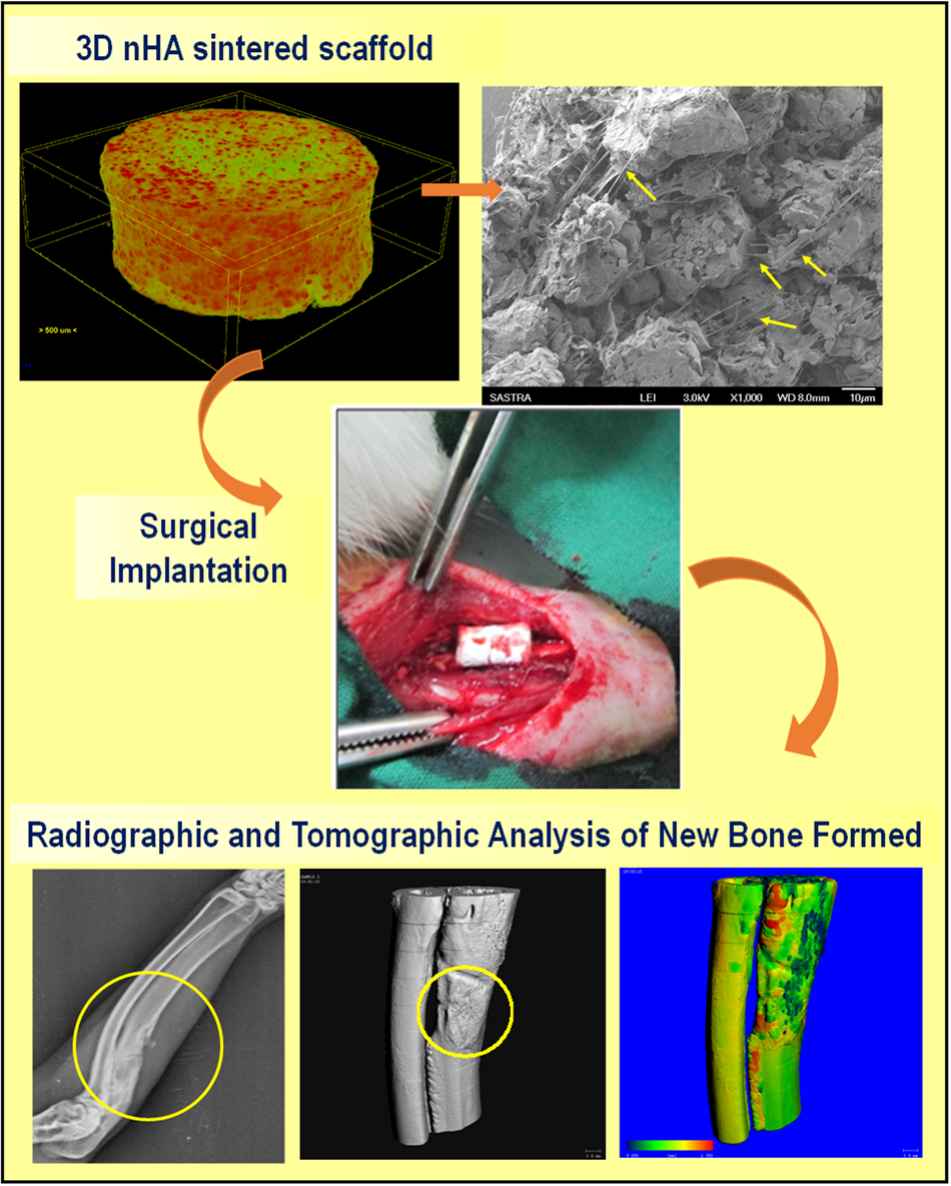
Graphical abstract

Graphical abstract

## Introduction

Large critical-size segmental bone defects fail to initiate the regular healing cascade to repair itself [1]. Complete shattering or absence of bone in such defects remains challenging in orthopedics and repair relies on bone graft transplantation [2, 3]. Autografts are the only available gold standard therapy for critical bone defects, but shortcomings such as donor site morbidity, limited availability and hemorrhage limits its use in bone repair strategies [4]. There is an increasing demand in the search of synthetic bone alternates as allografts has major shortcomings of immune complications, disease transmission and poor neovascularization. Advent of tissue engineering strategies expedites the construction of biomimetic bone substitute that are either equivalent to mineralized bone matrix or interconnected porous microstructure of trabecular bone tissue. Such porous biomimetic scaffold should demonstrate osteoconduction, osteoinduction and osteointegration with the native bone in addition to promoting tissue mineralization [5–8].

Various remedial strategies that effect the biomineralization include administration of proteins like bovine serum albumin (BSA), recombinant proteins, osteogenic factors or drugs like antibiotics that subsequently kindle endogenous regenerative potential or alleviate pain and prevent infection in challenging bone defects [9, 10]. Proteins play major role on precipitation of calcium phosphates and BSA interaction with nHA has been reported to induce biomineralization [11]. Integrating osteoinducting components in biomaterial encourages the bone regeneration through osteogenic differentiation of local mesenchymal stem cells. Bone morphogenetic proteins (BMPs) mainly BMP-2 and BMP-7 have been used widely in trauma and orthopaedic surgery as these cytokines enhances osteogenesis [12]. However, requirement of supra-physiological dose associated with undesirable side effects such as uncontrolled tissue growth, ectopic bone formation, inflammation and threat of carcinogenicity are the major clinical barriers [13]. Unlike BMPs, calcium-phosphate ceramics mimics biominerals found in hard tissue offering better biocompatibility, osteoconductivity and osteointegrating properties in addition to its biodegradability [14, 15]. Though no biomaterials form bone *de novo*, super saturation of calcium and phosphate ions at macro- and micro-porous structures of scaffold offers material–directed osteoinduction by facilitating cell infiltration and nutrient supply [16]. In general, calcium phosphate based biomaterials are osteoconductive, while some porous synthetic HA and similar materials demonstrate osteogenic potential at nonbony sites have been reported as osteoinductive [16]. Further substrates with porous architecture on its surface also promote mechanical intertwining with adjacent tissue to improve mechanical integrity of the implant, demonstrating osteointegration [17].

Nanohydroxyapatite (nHA) is the major inorganic component of bone exhibiting hexagonal crystal structure, biocompatibility, osteoconductivity, higher stability, and larger surface area with osteointegration property [18]. Many synthetic approaches are being used to synthesize hydroxyapatite of various shapes such as nanofibers, nanoparticles, nanorods and nanoflakes [8, 19, 20]. However, brittle nature of ceramics with unsatisfactory tensile strength and fracture toughness do not recommend the use of osteoconductive hydroxyapatite for load-bearing applications [21]. To circumvent the limitations of hydroxyapatite, bio-composites have been developed by combining benefit of polymer toughness and hydroxyapatite strength. Further inclusion of inorganic phase into the polymer phase would also maintain the neutral pH during the acidic by-products of the polymers [22]. Although the mechanical and biological properties of composites is relatively enhanced than pristine polymers, fabricating scaffolds with interconnected porous characteristics still remains challenging. Our group has developed protein loaded 3D mesoporous composite sintered scaffolds using a blend of poly(hydroxybutyrate) and poly(ɛ-caprolactone) (PHB–PCL) with inorganic component (nHA) and showed the presence of protein-mineral interface similar to native bone tissue [23]. The developed porous composite microspheres thus formed were sintered to 3D scaffolds by dynamic solvent/non-solvent sintering, as thermal sintering mechanism fails to sinter thermally stable inorganic materials and denature heat-labile proteins [23]. Dynamic solvent sintering is based on fractional solubility, where polymer dissolution and precipitation are well controlled by solvent/non-solvent composition, ratio and sintering duration. The dynamic solvent wets microsphere surface, swells the polymer chain, loosen, and subsequently interact with polymer chains of adjacent microsphere. The solvent is more volatile than non-solvent which causes precipitation of polymer chains, interactions such as locking, entanglement, and intertwining to become permanent with precipitation, and eventually leading to microsphere sintering. The sintering process allows preloading of nHA within the polymeric scaffold for sustained release [24]. Interestingly, inorganic nHA and organic polymeric blend PHB-PCL in the composite scaffold provide multiple cues such as biocomposition, meso-micro multi-scale porosity, compressive strength and nHA-protein interface.

In the present study, the composite phase-induced-mesoporous microspheres scaffold was evaluated using in vitro and in vivo methods to test the osteogenic regenerative potential using human osteoblast-like cells (MG63 cells) and rabbit ulnar segmental defect model, respectively. Rabbit was chosen as animal model as its mineral density and fracture toughness of mid-diaphyseal bone resembles human [25]. Cell-matrix interaction, cell proliferation and mineralization potential of the sintered scaffold were evaluated using MG63 cell lines. Cylindrical scaffolds (5 × 10 mm) were implanted following segmental (10 mm) defect in rabbit ulnar bone and the defects without any implantation served as control. Radiography (4, 8 and 12 weeks) and µ-computed tomography (12 weeks) analysis were performed to determine the bone tissue formation whereas histological studies such as hot Stevenel’s blue and van Gieson’s picrofuchsin staining were carried out to determine the maturation of bone tissue after 12 weeks.

## Materials and Methods

### Materials

Polymers such as poly(hydroxybutyrate) of MW 550 kDa, poly(ɛ-caprolactone) of MW 300 kDa and poly(vinyl alcohol) of MW 125 kDa were obtained from Lakeshore, USA, Good Fellow, UK and SD Fine Chemicals Limited, India respectively. Human osteoblast-like cells (MG63 cells) were procured from National Centre for Cell Science (NCCS), India. MEM media, Dulbecco’s phosphate buffered saline (PBS), Trypsin, penicillin–streptomycin and fetal bovine serum (FBS) were procured from Gibco, India.

## Methods

### Fabrication of composite sintered scaffold with Nanohydroxyapatite-protein interface

Composite porous sintered scaffolds were fabricated by incorporating the synthesized nHA into polymeric blend to prepare composite porous microspheres and further sintered in solvent/non-solvent system to form 3D scaffolds, as previously reported [23]. Briefly, nHA was synthesized using calcium nitrate tetrahydrate (Ca(NO_3_)_2_^.^4H_2_O) and di ammonium hydrogen phosphate ((NH_4_)_2_HPO_4_) as precursors by wet chemical precipitation. Porous composite microspheres were prepared by single emulsion (o/w) technique. The organic phase, polymeric blend of PHB and PCL (1:1) dissolved in chloroform and dispersed with nHA was continuously stirred in PVA as aqueous phase. The emulsion was centrifuged, washed with distilled water and the microspheres were obtained after 24 h of lyophilization. Sintered scaffolds (SS) were obtained using chloroform and ethanol as solvent and non-solvent (1:3) respectively. The microspheres were wetted, vortexed for 10 s and filled into cylindrical mold of specific dimension and dried in vacuum. Protein sintered scaffolds (P-SS) were prepared similarly with the inclusion of aqueous BSA (0.6%) solution in the solvent/non-solvent system.

### Micro-Computed Tomography (CT)

The composite microsphere sintered scaffold was scanned in air using high-resolution micro-CT scanner (Skyscan 1176, Bruker, Belgium) as reported previously [26]. No filters were used and the scanning parameters were 9 µm resolution, 40 kV voltage, 600 µA current, 465 ms exposure time, 0.3° rotation around 360° with 657 projections. Skyscan NRecon software was used for 3D reconstruction of the X-ray projections and further processed in Skyscan CT Vox for visualization.

### In vitro studies using MG63 cells

#### Cell maintenance and seeding

Human osteoblast-like cells (MG63 cells) were cultured in MEM supplemented with 10% FBS and 1% penicillin/streptomycin solution as monolayer in 5% CO_2_ incubator at 37 °C. Prior to seeding cells at a density of 5 × 10^4^ cells/well in 96 well plate, the scaffolds were UV sterilized on both sides for 1 h. Culture media was changed every 24 h in TCPS and scaffolds.

#### Cell proliferation by DNA quantification assay

Proliferation of cells that were seeded on TCPS and scaffolds (SS and P-SS) was determined by DNA quantification using Hoechst fluorimetric evaluation [27]. Shortly, after 7, 14 and 21 days of culture, well plates and scaffolds were washed with phosphate buffered saline (PBS) and distilled water was added. The cells were lysed to release the DNA by repeated freeze-thaw cycles. After three cycles, samples (50 µL) were mixed with Tris–NaCl–EDTA buffer and 100 µL Hoechst stain was added. The content was mixed well and fluorescence was measured at excitation (355 nm) and emission (460 nm) wavelengths using Tecan. The cell number was assessed by calibration curve prepared for up to 3.5 × 10^6^ cells density.

#### Alkaline phosphatase activity

The phenotypic bone marker alkaline phosphatase activity was measured based on hydrolysis of p-nitrophenyl phosphate to p-nitrophenol [28]. The alkaline phosphatase activity of MG63 cultured on SS, P-SS and TCPS was evaluated using alkaline phosphatase substrate kit (Bio Rad, USA) as per manufacturer’s protocol. At pre-determined time points (1, 7, 14 and 21 days), the cells were lysed by adding 1% Triton X-100 to each well. About 400 µL of freshly prepared substrate solution was mixed with 100 µL of cell lysate and incubated for 30 min at 37 °C. Sodium hydroxide (0.4 M) was added to terminate the reaction and absorbance read at 410 nm using multimode reader (Infinite 200 M, Tecan, USA).

#### Cell adhesion and morphology

The adhesion of cells on the scaffolds and its expansion were qualitatively assessed using scanning electron microscopy. After predetermined duration of culture (1, 14 and 21 days), scaffolds were washed with PBS and fixed using 4% glutaraldehyde at 4 °C for 48 hours. Fixed scaffolds were washed with PBS and dehydrated with graded alcohol (50 to 100% v/v), air dried and stored under vacuum. The dried scaffolds were sputter–coated with gold and observed using FE–SEM at accelerating voltage of 3 kV.

#### Gene expression analysis

The expression profile of alkaline phosphatase and osteopontin genes in osteoblasts cultured on SS and P-SS was determined using TCPS as control. After time intervals of 1, 7, 14 and 21 days, total RNA was extracted from the osteoblasts cultured constructs by adding Trizol as reported earlier [27]. In brief, chloroform (0.2 mL) was added to trizol extracted RNA, centrifuged and stabilized in 70% ethanol. The RNeasy minispin kit was used for isolating purified RNA as per manufacturer’s protocol and suspended in RNAse-free water. The reverse transcription kit was used for cDNA synthesis and subjected to realtime RT-PCR (Eppendorf AG22331, Germany) using SYBR green for gene expression analysis. Table 1 shows the nucleotide sequences of primers used in this study. The relative fold change were determined by 2^-ΔΔCT^ method and normalized with GAPDH as house-keeping gene [29].

**Table 1:** Forward and reverse primers sequences used for RT-PCR

Genes	Forward primers	Reverse primers
GAPDH	5′-AGGTCGGAGTCAACGGATTT-3′	5′-ATCTCGCTCCTGGAAGATGG-3′
Osteopontin	5′-ACTGATTTTCCCACGGACCT-3′	5′-CTCGCTTTCCATGTGTGAGG-3′
Alkaline Phosphatase	5′-GTCAGTGGGAGTGGTAACCA-3′	5′-ACATGTACTTTCGGCCTCCA-3′

### In vivo bone regeneration

#### Rabbit Ulnar non-union defect and radiographic analysis

**Fig. 1:**
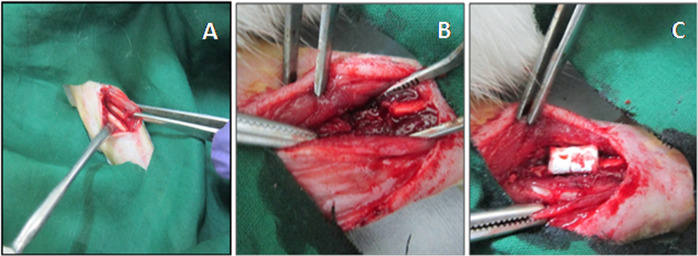
Implantation of microsphere sintered scaffold in rabbit ulnar model. **A** The radial and ulnar bones were exposed. **B** 10 mm defect in ulnar bone. **C** Scaffold implanted at the defect site

The ulnar segmental defects were created in 12 New Zealand white male rabbits (3–5 kg) as reported with minimal modifications [29]. All the surgical procedures and surgical care was approved by Institutional Animal Ethical Committee (IAEC) of SASTRA University (270/SASTRA/IAEC/RPP). The animals were divided into three groups: control, sintered scaffolds (SS) and protein loaded sintered scaffolds (P-SS). The animal was anesthetized by administration of Ketamine (22 mg/kg)/Thiopentone sodium (30 mg/kg) and Xylazine (10 mg/kg) through intramuscular route. The surgical site, right forelimb was shaved, prepped and sterilized with betadine and 70% ethanol. The ulna bone was exposed by incision over the ulnar region and soft tissue was resected away from the bone (Fig. 1A). An ulnar segment of 10 mm was removed using a bone cutter while simultaneously irrigating with saline. SS or P-SS scaffolds (diameter 5 mm and length 10 mm) was placed on the defect site and layers of tissue was closed using degradable sutures (Fig. 1B, C). The wound was closed without implantation in control group animals. No external fixation was required to secure the ulnar bone, as radius provides natural splint for ulna. Animals were fed *ad-libitum* and permitted to walk freely within their cages after surgery. The segmental defects were observed by clinical radiographic analysis on the day after surgery and healing was monitored at predetermined time intervals (4, 8 & 12 weeks) during the study period.

#### Micro-CT analysis

The morphology of new bone formed at defect site was assessed after 12 weeks using high-resolution X-ray micro-CT (Scanco Medical, Switzerland). Scanning was performed using X-ray beam energy 70 kV and 114 µA X-ray intensity. Around 500 projections were reconstructed using cone-beam convergence/beam projection algorithm based software. Bone morphometric analysis such as Bone volume fraction (BV/TV %), Specific bone surface (BS/BV mm^−1^), Trabecular thickness (Tb.Th. mm), Trabecular separation (Tb.Sp. mm), Degree of Anisotropy (DA), and Bone mineral density (mg HA/ccm) were performed in the defect region as region of interest (ROI) to evaluate bone regeneration.

#### Histopathological analysis

After 12 weeks post-surgery, animals were euthanized and the forelimbs were collected and processed for histology as reported earlier with modifications [30]. Briefly, the soft tissues were resected away from the bones and cut at around 2 cm away from the ends of implanted scaffolds. The radial and ulnar bones with new tissue formed were fixed in 10% formaldehyde, dehydrated using graded ethanol concentrations (70–100%), embedded in methylmethacrylate (MMA) and allowed to polymerize. After polymerization, the tissue embedded PMMA blocks were sectioned (70–100 µm) using a linear precision saw microtome (ISOMET 5000, Bueler, GmbH). The sections were fixed to a glass slide, ground and surface was polished using variable speed grinder polisher (ECOMET 3000, Bueler, GmbH). Hot Stevenel’s blue and van Gieson’s picrofuchsin staining were observed under trinocular transmission light microscope (Nikon E600) and photomicrographs were captured by camera (Nikon DS Ri1).

### Statistical analysis

Data have been expressed as mean ± standard deviation of values obtained from three independent experiments. For cell studies, two-way analysis of variance (ANOVA) with Tukey post-hoc test was carried out to determine statistical significance between TCPS, SS and P-SS. SPSS 15.0 statistical software was used with *p* < 0.05 as criterion for statistical significance.

## Results

Spontaneous bridging of large segmental bone defects remain challenging in clinics, as it demands the intervention of tissue-engineered constructs for union [31]. Hence, the present study focused on the development of multicomponent composite scaffold to harness physiological healing potential. Nanohydroxyapatite embedded protein microspheres were sintered and the multicomponent composite scaffold with multi-scale porous microstructures similar to composition, structure and mechanical strength was developed. The functional efficacy on bridging the critical-sized defects in the non-union rabbit ulnar model was further investigated.

### Nanohydroxyapatite-protein enriched sintered scaffold

**Fig. 2:**
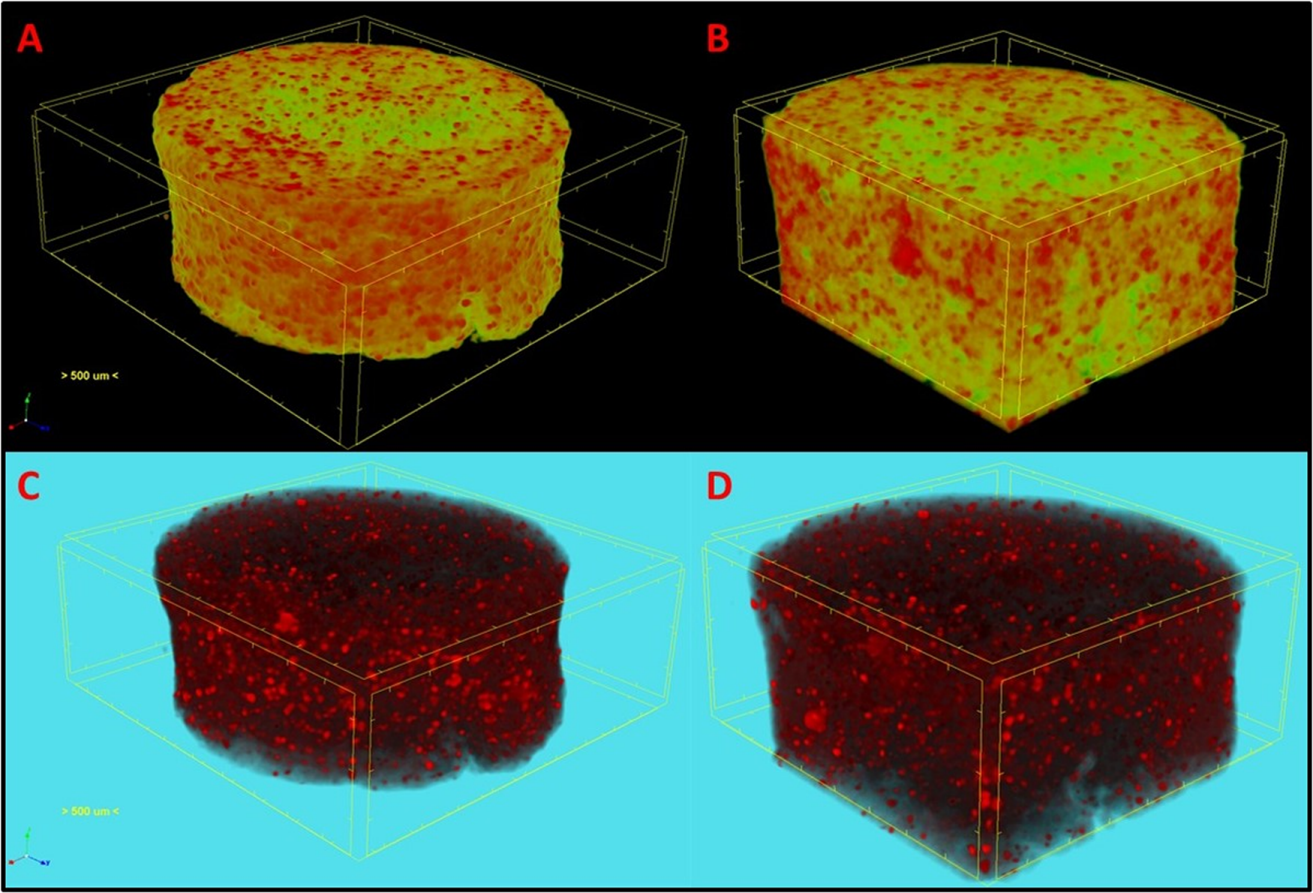
Three-dimensional reconstructed micro-computed tomographic images of composite microspheres sintered scaffold showing homogenous distribution of nHA particles (red) in green matrix (**A**) surface and (**B**) longitudinal sections. Threshold adjusted to visualize nHA dispersion in transparent scaffold (**C**) whole scaffold and (**D**) longitudinally sliced faces

Micro-CT analysis has been used as tool to delineate the pattern of nanohydroxyapatite particles distribution in three-dimensional polymeric scaffolds [26]. Figure 2 shows 3D reconstructed X-ray micro-CT images of composite microspheres sintered scaffold. Micro-CT distinguishes the compositional differences in scaffolds that enables visualization of high dense bioceramic nHA particles distribution in polymeric matrix. Figure 2A, B showed the distribution pattern of nHA particles (red) throughout the scaffold represented as green porous matrix. The high dense inorganic nHA particles were delineated by varying the threshold and the distribution was demonstrated (Fig. 2C, D). Further, the pattern of distribution in longitudinal sections (Fig. 2B, D) and surface was observed thereby depicting the presence of bioceramic throughout the scaffold.

### Proliferation and alkaline phosphatase activity of MG63 cells in vitro

**Fig. 3:**
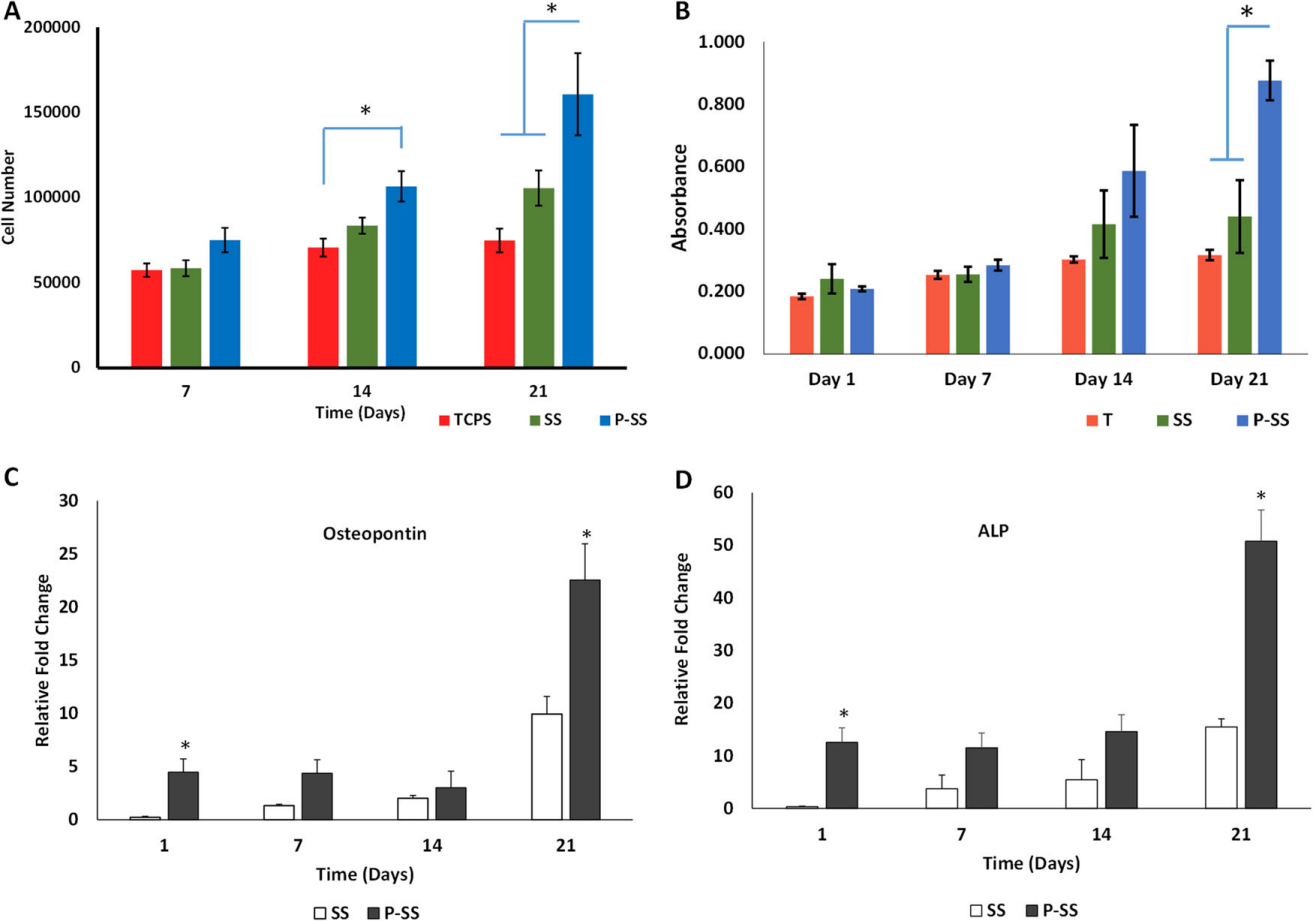
**A** Cell proliferation assayed by DNA quantification in sintered scaffold (SS), protein-loaded SS (P-SS) and TCPS (*p < 0.05). **B** Alkaline Phosphatase activity of MG-63 cells cultured in sintered scaffold (SS), protein-loaded SS (P-SS) and TCPS (T); Gene expression of (**C**) Osteopontin and (**D**) Alkaline Phosphatase (ALP) with MG-63 cells cultured on sintered scaffold (SS), protein loaded SS (P-SS) for 21 days (*p < 0.05). (*p < 0.05)

**Fig. 4:**
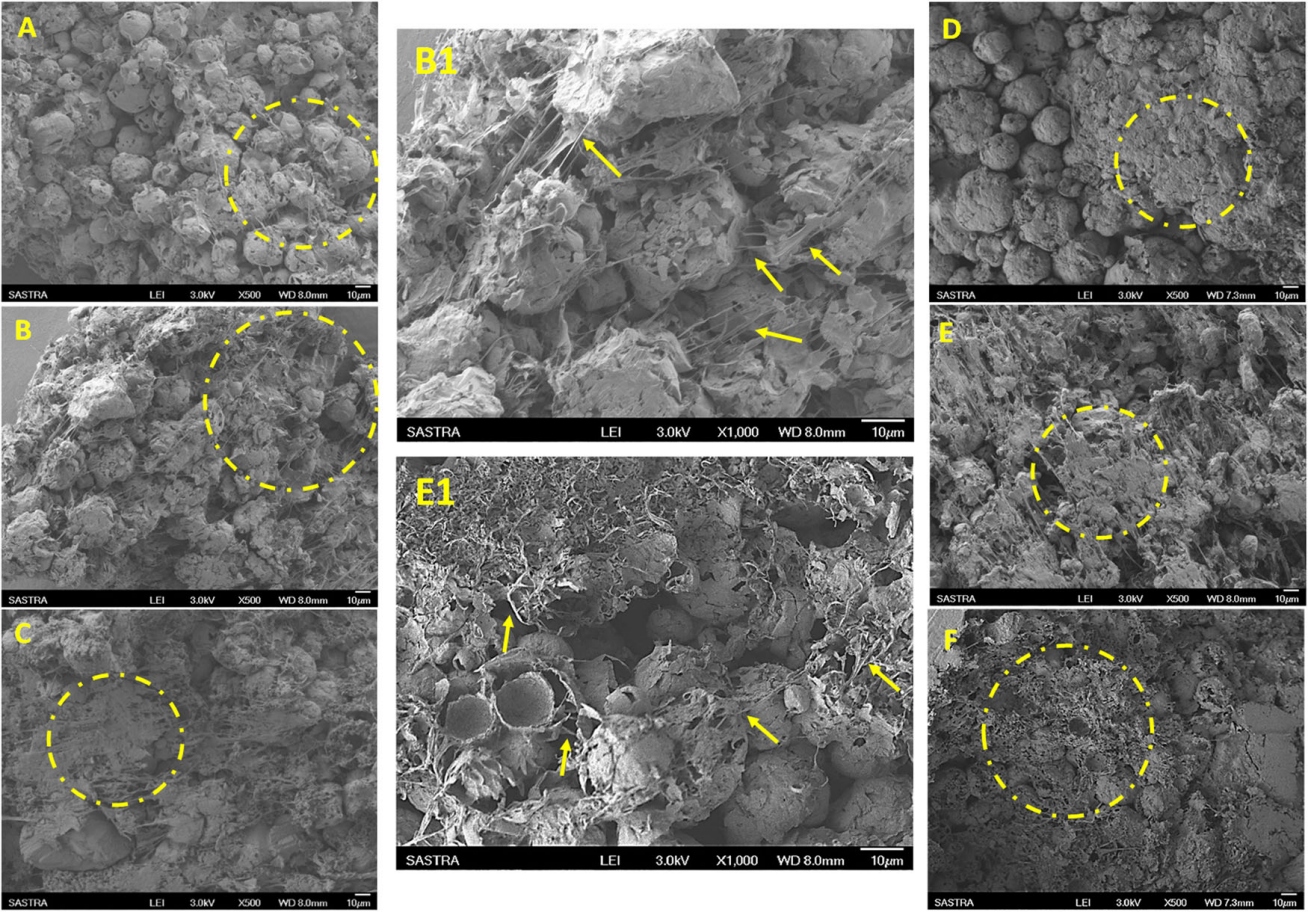
Scanning electron micrographs of cells cultured on SS after (**A**) 1 day. (**B**) 14 days. (**B1**) Higher magnification at 14 days (**C**) 21 days and on P-SS scaffolds after (**D**) 1 day. (**E**) 14 days; (**E1**) Higher magnification at 14 days and (**F**) 21 days of culture. Circles indicate cell bodies and arrows indicates cell filopodia

Cell number was significantly higher at days 14 and 21 of culture on nanohydroxyapatite-protein enriched sintered scaffold compared to TCPS, which increased further to significantly higher in protein scaffold compared to both TCPS and scaffold devoid of protein (*p* < 0.05). High proliferation rate of osteoblast-like MG 63 cells on protein-loaded porous scaffolds confirms the cytocompatibility and regenerative potentials of the developed composite (Fig. 3). Significant increase in osteogenic potentials was confirmed with increased alkaline phosphatase activity in MG 63 cells over 21 days in P-SS group. In vitro DNA quantification and alkaline phosphatase (ALP) assays revealed that the protein scaffolds supported osteoblast proliferation and mineralization significantly higher than scaffolds devoid of protein and TCPS (Fig. 3A, B). Scanning electron micrographs of osteoblasts cultured scaffolds demonstrated osteoblast adhesion on both SS and P-SS after one day (Fig. 4A, D). The cell spreading, colonization and filopodial extension across the porous voids with the accomplishment of cell-matrix interaction was evidenced in both protein and void scaffolds after 14 days (Fig. 4B, B1, E, E1). The cell coverage over the scaffolds progressed towards complete spreading after 21 days, which could be attributed to the composition, multi-scale porosity that serves as multiple cues to promote cell proliferation (Fig. 4C, F).

### Quantitative real-time PCR

The mRNA levels of osteopontin and ALP in MG63 cells seeded on scaffold revealed significant fold change in P-SS compared to SS (Fig. 3C, D). Osteopontin and ALP expression was relatively increased 19 and ~38 folds in P-SS than SS in day 1 whereas 2.2 and 3.2 folds in day 21, respectively. These results indicate that protein loaded scaffolds promoted osteogenesis than bare scaffolds.

### In vivo bone regeneration

**Fig. 5:**
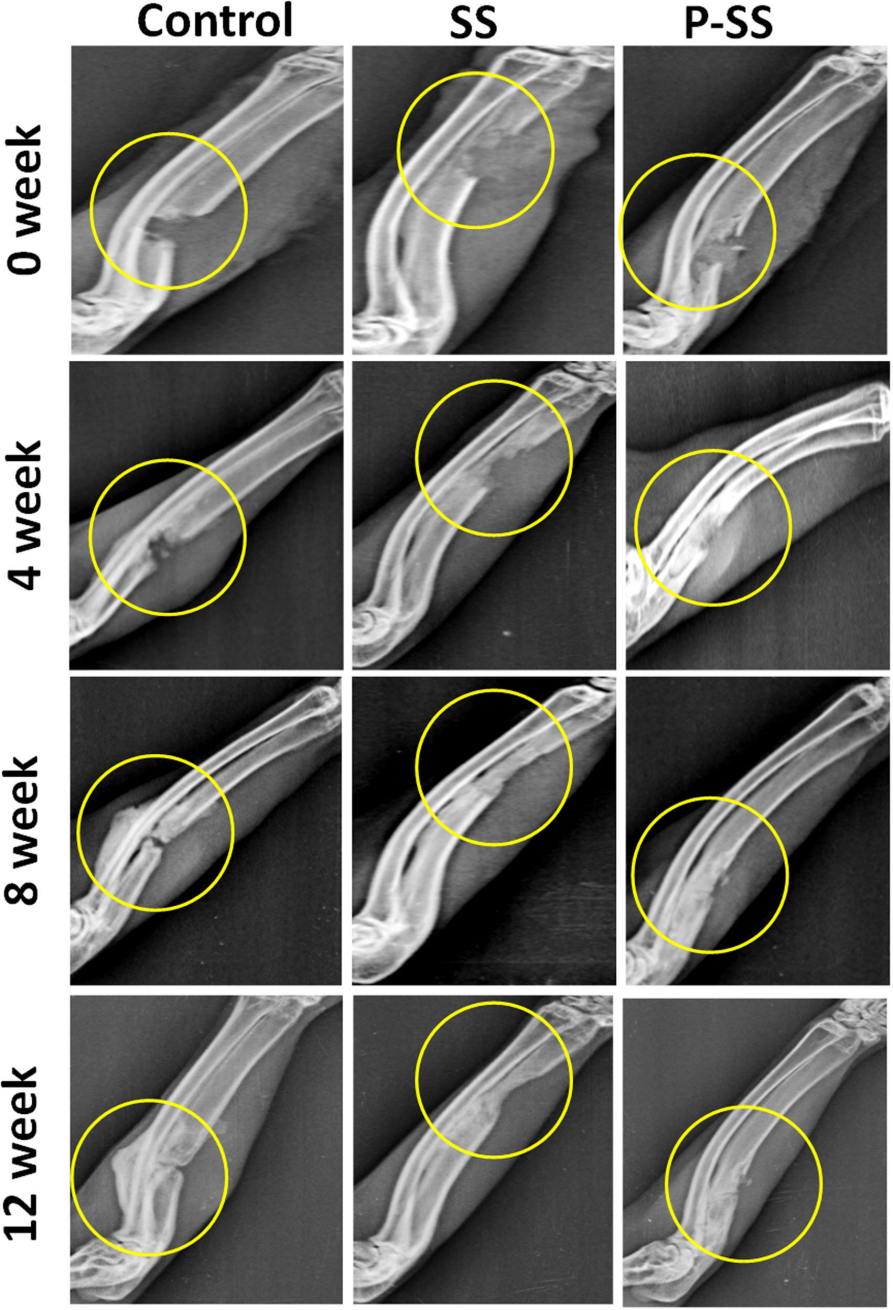
Radiographic analysis of control, SS and P-SS treated rabbit ulnar bone defects after 0, 4, 8, and 12 weeks of surgery. Circles indicate defect regions with new bone formation

Rabbit ulnar defects were filled with cylindrical scaffolds (5 × 10 mm) and covered the denuded medial and distal ends following segmental (1 cm) defect. Defects closed without implantation served as control. After surgery (0 week), the defect region was clearly visible in the ulna bone of the three groups. The radiographs post-surgery showed the healing of defect created in ulnar bone for 12 weeks in control and sintered scaffolds (Fig. 5). Partial thickening of new bone in P-SS group denoting early signs of healing was observed after 4 weeks whereas minimal growth was seen in SS and control animals. Complete fusion of new bone with the medial and distal ends of defect was significantly evident in P-SS filled defects after 12 weeks. Whilst, undesired sclerotic-like tissue formation was observed in control group from 8 weeks.

### Micro CT analysis

**Fig. 6:**
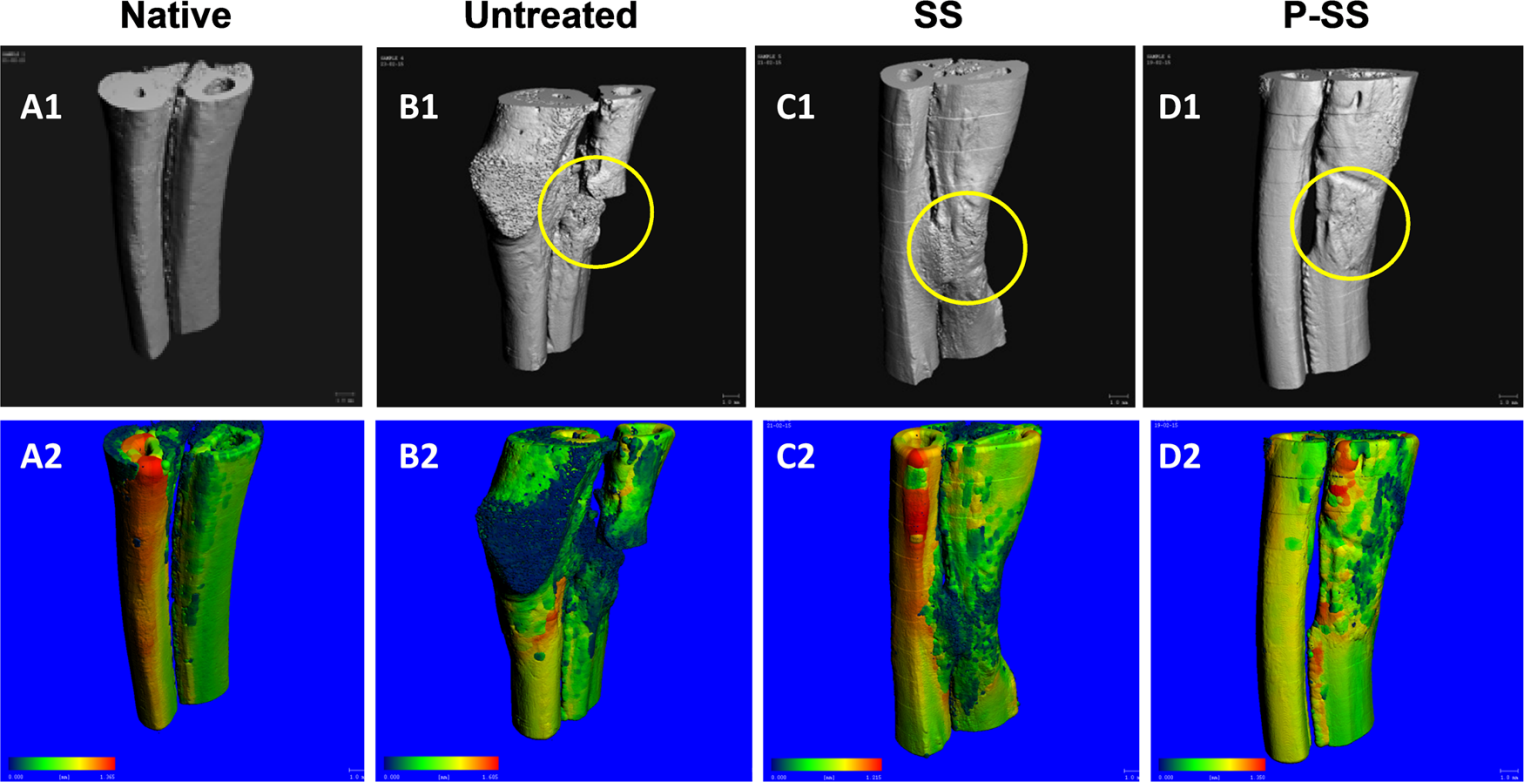
Micro-CT analysis of (**A**) native, (**B**) Control and implanted groups (**C**) SS and (**D**) P-SS after 12 weeks of surgery

The tissue morphogenesis and mineralization of new reparative tissue formed in the defect site was analyzed in sintered scaffold groups and compared with control and native bone. Figure 6A–D1 showed the morphology of new bone with the neighboring host tissue in the greyscale images. Micro CT images of segmental bone showed complete fusion of new bone with the adjacent host bone after 12 weeks without affecting the structural architecture of bone in P-SS implantation (Fig. 6). However, fusion with radial bone in SS – filled group showed lack of defined bone growth that may lead to distortion in functional properties. Radiography (4, 8 and 12 weeks) and µ-computed tomography (12 weeks) analysis evidenced directional bone tissue formation by bridging the gap at the defect site in both protein-loaded (Fig. 6D1) and protein devoid scaffold groups (Fig. 6C1). Figure 6A-D2 investigated the thickness variation of neo-tissue with surrounding host tissue represented in 3D color images. The blue color in neo-tissue of control group (Fig. 6B2) denotes lesser value for thickness variation. Interestingly, the more green color in neo-tissue of protein scaffold (Fig. 6D2) and native bone (Fig. 6A2) represented the similarity in thickness variations. In addition, the bone morphometric parameters were quantified to compare the features such as trabecular thickness, separation, trabecular volume and bone volume on the reconstructed images. Table 2 showed that the new bone formed in P-SS showed higher Trabecular thickness (0.6776 mm), separation (1.043 mm), mean/density of bone volume (1040.56 mg HA/cc) compared to control and sintered scaffold group.

**Table 2:** Bone morphological parameters quantified using microCT in the volume of interest after 12 weeks of surgery

Morphometry	Native Bone	Control	SS	P-SS
Trabecular thickness (Tb.Th) mm	0.7784	0.6317	0.62	0.6776
Trabecular separation (Tb.Sp) mm	0.9752	0.4975	1.0295	1.0433
Bone volume fraction (BV/TV) %	0.7502	0.738	0.7316	0.6978
Bone surface density (BS/BV) %	1.7431	4.1274	2.6335	2.5405
**Mean/density (mg HA/cc)**				
BV (Material)	1068.1196	990.7358	1019.2212	1040.562
Degree of Anisotropy	2.3548	1.5219	1.8473	1.8338

*SS* Sintered scaffold, *P-SS* Protein loaded sintered scaffold

### Histopathological analysis

**Fig. 7:**
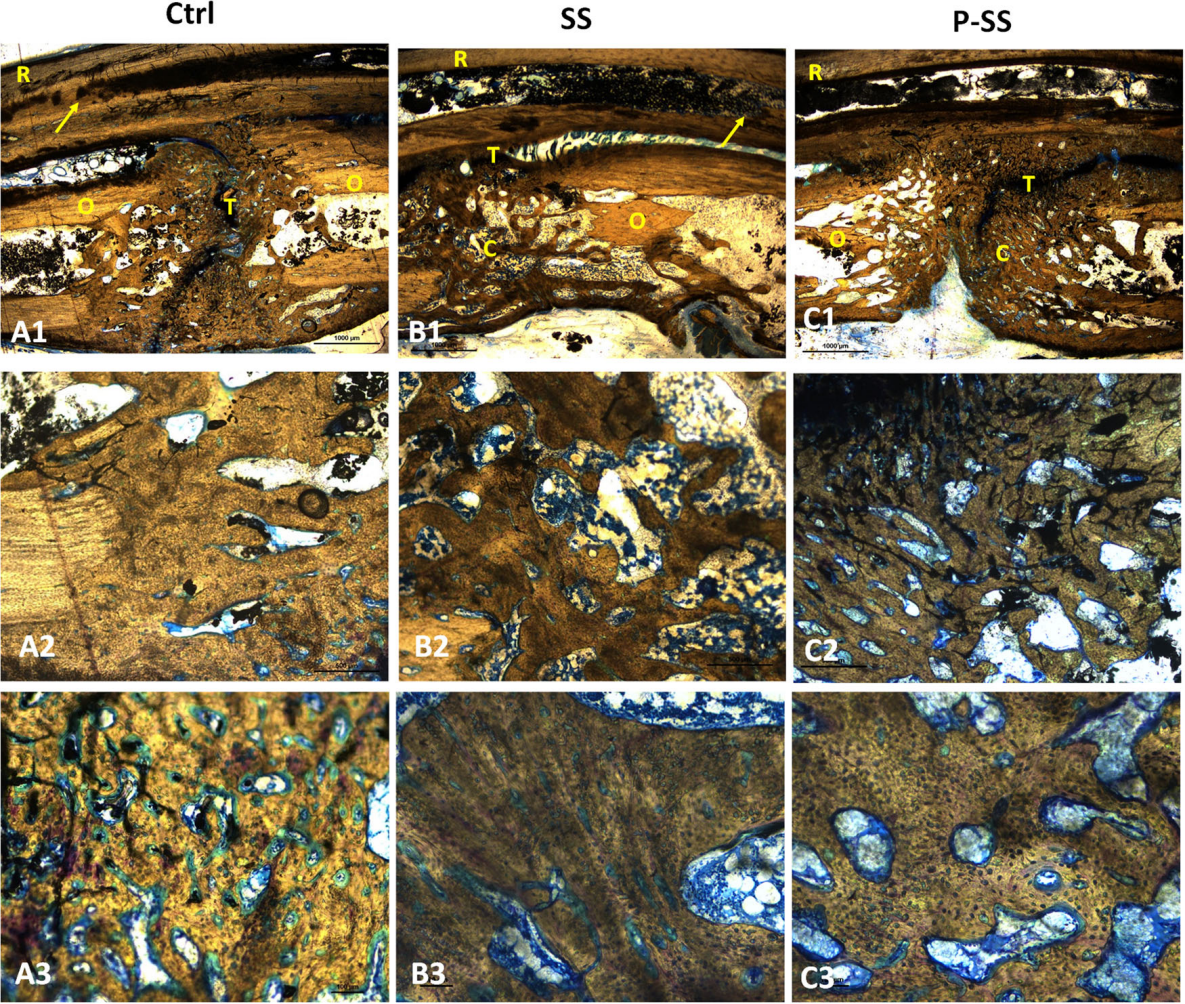
Histology analysis by Stevenel’s blue and van Gieson’s picrofuchsin stains after 12 weeks on (**A**) control; (**B**) SS and (**C**) P-SS, (**A1**, **B1** & **C1**) showing the interface between old and newly formed bone (Scale bar: 1000 µm); (**A2**, **B2** & **C2**) represents features at low (Scale bar: 500 µm) and (**A3**, **B3** & **C3**) high magnifications (Scale bar: 100 µm) respectively. (R Radial bone (old bone); O Osteoid (young bone); C Calcified (new bone); T Tricalcium Phosphate; arrows indicate sites of neo-tissue fusion with radial bone)

In all the three groups, no sign of inflammation or bone necrosis was observed at the surgery site. Histology by hot Stevenel’s blue and van Gieson’s picrofuchsin staining confirmed enhanced bone maturation in scaffold groups while presence of osteoids/immature bone was observed in control after 12 weeks (Fig. 7). The region at interface between old bone and newly formed bone shows fusion of reparative bone tissue with adjacent radial bone (R) in control (Fig. 7A1). Figure 7C1 showed the fusion of ulna bone, while the radial bone (R) remained alone without fusion with newly formed bone in ulna. Regenerated bone showed yellowish-green stained osteoid tissues surrounding red-stained mineralized bone tissue, with the cohort of newly differentiating osteogenic cells contributing actively in initial bone formation [32]. The higher magnification of regenerated region in protein sintered scaffold group exhibited rosettes of osteoblasts and osteoclasts as foci of bone remodeling were observed (Fig. 7C3). Further, ossified bone matrix, osteoid and cells were observed in the pores and grooves of control and protein sintered scaffold groups [30].

## Discussion

Engineered scaffold for bone regeneration should ideally facilitate osteoconduction, osteoinduction and osseointegration [30, 33]. The developed nHA-BSA enriched scaffolds constitute cylindrical nanopores of diameter ~34.34 nm and enhance the diffusion of nutrient supply for the cells [23, 34, 35]. The sintered scaffold has been developed with multi-scale porosity, nano-sized pores in porous microspheres and microporous architecture obtained by sintering of microspheres that ensures nutrient diffusion. Interconnect porous structures promote biological functions like cellular adhesion and deeper penetration of nutrients that generate more sites for nucleation of calcium and phosphate ions essential for calcification [36–38]. The positive influence of calcium and phosphate based substances such as hydroxyapatite and tricalcium phosphates on the osteogenic potential of scaffold is well known [39]. In addition, presence of BSA in the developed scaffold enhanced the cell proliferation and infiltration of cells in the scaffold as observed by DNA quantification (Fig. 3A). Several reports confirm the incorporation of nHA for strong binding with protein for sustained release and contribution towards cell migration, proliferation, mineralization and differentiation of native bone cells [12]. Enhanced ALP activity was quantified in protein loaded scaffold than the scaffold without proteins over 21 days in MG63 cells (Fig. 3B), which, is an early-stage marker for osteogenic differentiation [40, 41]. In addition to cellular proliferation and ALP activity, P-SS showed up-regulation of osteopontin and alkaline phosphatase genes in over 21 days (Fig. 3C, D). The lack of osteopontin expression at defective sites can lead to reduced bone formation as it plays important role in mineralization and its regulation [42]. Therefore, enhanced osteopontin expression can promote increase in bone mass and mineralization for faster bone healing at defect.

Endogenous stem cells especially mesenchymal stem cells (MSCs) play a crucial role in bone regenerative cascade towards progressive tissue healing [43]. Appropriate localization of these cells is of paramount importance and biomolecules such as serum albumin has been reported for its positive influence on stem cells [43]. Acellular scaffold with multiple cues including ideal structural, compositional and mechanical properties that mimics the extracellular matrix favors infiltration of reparative cells from the tissue adjacent to defect site and promote bone regeneration in vivo [44–46]. The developed sintered scaffolds with multiple cues have demonstrated regenerative and osteogenic potential, which contributed to the new tissue formation when implanted in rabbit ulnar bone segmental defect model. Figure 5 depicted the progressive regeneration of neo-tissue in the defect site in both sintered scaffold groups towards complete fusion of the ends after 12 weeks. On contrary, in control group the complete closure was not observed, sclerotic-like tissue formation occurred and fusion with adjacent radial bone was observed. Sclerotic tissue can be characterized by thickened, disorganized mineralization of the bone with coarse morphology [47]. The neo-tissue formation pattern was further confirmed by the 3D grey scale images of microCT analysis (Fig. 6C-D1). The 3D microCT colored images showed that the thickness variation of bone formed in P-SS was similar to native bone (Fig. 6A2, D2). The presence of microstructural and nHA-protein distribution as spatial and directional cues in P-SS group could be attributed to the directional formation of neo-tissue [48, 49]. Such spatial and directional pattern of reparative neo-tissue distribution was not observed in control group (Fig. 6B1). Stevenel’s Blue and Van Gieson’s Picrofuchsin differential tissue staining has been reported for histological analysis of in vivo calcified bone tissue regeneration induced by calcium phosphate based scaffolds [50, 51]. Histology investigation showed absence of host immunogenic response to the scaffold material after 12 weeks. The scaffold presented osteoconductive potential with organized new bone formation in the defect. The presence of bone remodeling cells, osteoblasts, and osteoid demonstrated that the new bone formation actively bridges the defect. Ossified bone matrix with organized bone and woven bone were clearly identified in P-SS group compared to SS and control group, thereby demonstrated the mineralized matured bone formation. The superior regeneration in P-SS could be attributed to the role of albumin-hydroxyapatite in the scaffold that imparts osteoinductive property and recruits stem cells for tissue healing [52]. Thus, nHA based multi-scale porous sintered scaffold exhibited the potential to infiltrate cells, enhance proliferation and promote osteogenic activity in vitro thereby contributing to the directional tissue formation with mineralization essential for new bone tissue regeneration in vivo.

## Conclusion

The developed protein sintered scaffold with meso-microporous structure attempts to closely mimic the native bone tissue multi-scale porosity, revealed the osteogenic potentials by increased proliferation rate and alkaline phosphatase activity in osteoblast-like cells over 21 days. Additionally, enhanced expression of osteopontin and ALP implicates the mineralization potential of the scaffold and can be correlated to the homogenous distribution of nanohydroxyapatite in the scaffold. The scaffolds induce new bone formation for healing in rabbit ulna segmental defect model within 12 weeks. X-ray radiographs revealed the bone formation pattern and microCT depicted the thickness variation of new tissue in addition to morphology. Histology analysis showed the presence of osteoblastic rosettes that represents osteoid formation confirming the induction of new bone formation. Thus, the developed composite matrices show osteoconductive, osteoinductive, and tissue formation properties that could be attributed to the nHA, nHA-protein interface, meso-microporous architecture and mechanical properties.
